# Irritable Bowel Syndrome: Prevalence and Risk Factors in Jazan Region, Saudi Arabia

**DOI:** 10.7759/cureus.15979

**Published:** 2021-06-28

**Authors:** Abdulelah M Arishi, Erwa E Elmakki, Othman M Hakami, Omar M Alganmy, Sultan M Maashi, Hamood K Al-Khairat, Yasir A Sahal, Abdulaziz A Sharif, Mohammed H Alfaifi

**Affiliations:** 1 Faculty of Medicine, Jazan University, Jazan, SAU; 2 Faculty of Medicine/Gastroenterology, Jazan University, Jazan, SAU

**Keywords:** irritable bowel syndrome, rome iv criteria, jazan, saudi arabia, risks factors

## Abstract

Background

The prevalence of irritable bowel syndrome (IBS) worldwide remains heterogeneous. In Saudi Arabia, there are insufficient studies on the prevalence of IBS among the general population, yet the prevalence of IBS in certain professional groups has been reported. This study was conducted to determine the prevalence of IBS and its associated risk factors in the Jazan Region of Saudi Arabia.

Methods

An online cross-sectional study was conducted from January to March 2020 in the Jazan Region of Saudi Arabia, using a multi-stage stratified sampling technique. The data were collected using a web-based validated Rome IV questionnaire. The Rome IV criteria are used to diagnose functional gut disorders, including IBS. Logistic regression analysis was used to determine the odds ratio (OR) with 95% confidence intervals (95% CI) for the selected risk factors.

Results

The survey included 1554 participants with an overall IBS prevalence of 16%. Women had a higher incidence of IBS than men (55.3% and 44.7%, respectively). IBS-mixed (32.66%) and constipation-predominant (32.25%) were the most common subtypes. In multiple regression analysis, female gender (OR = 1.503, p-value = 0.037), stress (OR = 2.386, p-value = 0.000), anxiety (OR = 1.943, p-value = 0.000), and tobacco smoking (OR = 2.093, p-value = 0.001) showed a statistically significant association with IBS.

Conclusions

The prevalence of IBS in the southwest region of Saudi Arabia is high. Female sex, tobacco smoking, stress, and anxiety are the major risk factors associated with IBS.

## Introduction

Irritable bowel syndrome (IBS) is a chronic functional gastrointestinal (GI) disorder that causes frequent distress in affected patients [1]. IBS is characterized by recurrent, chronic abdominal pain associated with changes in bowel habit frequency or stool consistency, in the absence of underlying organic lesions determined by endoscopy, laboratory, or radiological studies [2].

The main pathophysiology of its development is not fully understood. A combination of genetic predisposition, altered gut-brain interactions, visceral hypersensitivity, mucosal inflammation, and bowel microbial alternation may have contributed to IBS development [2,3].

The lack of objective diagnostic findings to identify IBS has restricted its diagnosis to the use of individual medical history. Currently, an IBS diagnosis is made using the Rome IV criteria [4]. The Rome IV criteria are as follows: recurrent abdominal pain on average at least one day per week during the previous three months associated with two or more of the following factors: pain related to defecation, change in stool frequency, and change in stool form or appearance. The Rome III criteria were updated to Rome IV in 2016. The Rome IV criteria have substituted “abdominal discomfort” with “abdominal pain,” which is more specific and clearer to patients than the vague term “discomfort” that was used in the Rome III criteria [5]. In addition, abdominal pain is not necessarily relieved by defecation; it may remain the same or even increase after defecation [6].

Globally, functional GI disorders affect almost 40% of the population worldwide. IBS accounted for 3%-5% of cases using Rome IV criteria, based on the recent Rome Foundation Global Study results conducted among 33 countries [7]. In a systematic review and meta-analysis study using Rome III and IV criteria, the global prevalence of IBS was found to be 9.2% and 3.8% in both IBS criteria, respectively [8]. There is a noticeable heterogenicity in epidemiological IBS studies that can be attributed to differences in methodology, culture, diagnostic criteria used, and unidentified other factors [9].

In the Kingdom of Saudi Arabia, most studies conducted on IBS prevalence were among school teachers [10], university students [11,12], and school children [13], with a prevalence range of 9%-40%. Most of these studies used previous versions of the Rome criteria. Moreover, they did not reflect the prevalence of IBS in the general population and yielded a greater variability in prevalence rates [14]. Recently, a few studies in Saudi Arabia used the new Rome IV criteria. One of these studies was conducted among board-certified physicians and surgeons, and found that 16.3% of participants met the IBS criteria [15]. Additionally, two studies were conducted using Rome IV among undergraduate students in Saudi Arabia, and reported an IBS prevalence of 15.8% and 8.8% [16,17].

Multiple risk factors have been strongly associated with IBS development. IBS appears more often in women and younger age groups [18]. Smoking also appears to be associated with IBS, but no significant association has been found based on a systematic review study [19]. Stress is a psychological risk factor that is strongly associated with IBS cases. All types of stress, whether physical, psychosocial, or psychological stresses, can influence IBS symptoms [20]. Anxiety, a psychological type of stress, is a common triggering risk for recurrent IBS symptoms [21]. Chronic stress also increases the severity of symptoms and leads to poor clinical improvement in patients with IBS [22].

Therefore, this study aimed to determine the prevalence of IBS and its associated risk factors in the Jazan Region, Saudi Arabia, using the recent Rome diagnostic criteria for IBS.

## Materials and methods

Study design and setting

A web-based cross-sectional study was conducted in the Jazan Region of Saudi Arabia between January and March 2020. Jazan Region is one of the administrative regions of Saudi Arabia and is located in the southwest area. It is bordered by the Red Sea from the west and the Republic of Yemen from the south. The total area of the region is estimated to be around 40,000 km^2^, and it has 13 sub-regions. The region is characterized by its varied topography, as it includes coastal, plain, and mountainous areas. According to the 2018 Saudi Arabia national population census, the region is populated by approximately 1.6 million people with a population density of 130 per km^2^. Jazan population is composed mostly of Arabian tribes, sharing the same ethnicity, religion, and language. 

Sampling technique

A sample of 1,990 participants was estimated for the purpose of this study. The sample size was calculated using Epi-Info software (version 7.2) developed by the Centers for Disease Control and Prevention (CDC). The following parameters were used for the population survey: population size of 1.6 million based on the Saudi Authority for Statistics-Population Surveys 2018; expected frequency of 16% based on a previous study using Rome IV criteria in Saudi Arabia [15]; 95% confidence level; 1.8% acceptable margin of error; and 25% non-response rate.

A multi-stage stratified sampling technique was used. The directorate of health affairs of the Jazan Region stratifies the 13 sub-regions of Jazan into six sectors: northern, southern, eastern, western, central, and major sectors. A representative subregion was randomly selected from each sector, including Baish, Samtah, Faifa, Sabya, Abu Arish, and Jizan. Finally, a probability proportionate to the size sampling technique was used to determine the number of participants for each representative subregion. Subjects were recruited from the primary healthcare centres registrar of each determined subregion. After recruiting participants by simple random sampling, the questionnaire was sent through social networking applications to each registered phone number of participants (e.g., Telegram, WhatsApp, and phone SMS). Individuals who self-reported alarming symptoms or organic diseases similar to IBS were excluded from the study. For inclusion in the study, online participants were required to be 18-69 years old, and able to read and write in Arabic.

Data collection 

Data were collected using a web-based questionnaire through Google Forms. The questionnaire included six sections. The first section collected socio-demographic information. The second section included a self-reported list of alarming symptoms and organic diseases that may mimic IBS as an exclusion criterion. Alarming symptoms included: history of rectal bleeding or melena; unexplained recent weight loss; family history of inflammatory bowel disease or colorectal cancer; night-time pain; or diarrhea. The list of self-reported organic diseases included inflammatory bowel disease (Crohn’s disease or ulcerative colitis), colorectal cancer, celiac disease, and lactose intolerance. Individuals who self-reported the presence of any alarming symptoms or known organic diseases were excluded from the IBS diagnosis. The third section used questions about IBS adopted from the validated Rome IV questionnaire for adults. The Rome IV questionnaire evaluates several functional GI disorders, including IBS [4]. A license agreement was obtained from The Rome Foundation for using the Rome IV diagnostic questionnaire with an Arabic translation. The Rome IV questionnaire also subclassified IBS participants into the following four subtypes based on the Bristol Stool Scale: constipation-predominant IBS, diarrhoea-predominant IBS, mixed IBS, or unclassified IBS subgroups. The fifth section included questions on certain addictive substances and other risk behaviours common in Jazan Region, such as khat chewing (a commonly chewed addictive green leaf in Jazan Region), tobacco smoking, and caffeinated drink consumption. The last section contained questions regarding psychological risk factors known for IBS, which are mainly stress and anxiety. Stress and anxiety questions were obtained from the Arabic-validated version of the Depression, Anxiety, and Stress Scale 21 (DASS-21), developed by Lovibond and Lovibond [23]. The DASS-21 uses a 4-point Likert scale to rate participants’ perceptions. A DASS-21 score of ≥8 for anxiety and ≥15 for stress were considered as cut-off values for having these conditions.

Data analysis

Statistical analysis was performed using Statistical Package for Social Sciences (SPSS) version 25 (SPSS Inc., Chicago, IL, USA). Data analysis involved descriptive statistics and inferential statistical techniques. The primary outcome was the prevalence of IBS and its subtypes among the Jazan population in Saudi Arabia. The secondary outcome was the correlation of sociodemographic factors, khat chewing, tobacco smoking, caffeine consumption, stress, and anxiety with IBS; using Pearson's chi-square test for categorical variables, Student's t-test for continuous variables, and multiple regression analysis for selected risk factors that showed a significant association in bivariate analysis. Associations were considered statistically significant when p-value < 0.05.

Ethical consideration

Participants were informed that they had the right to withdraw from the study at any time, their information would be kept anonymous, and the data collected would only be used for scientific purposes. The Institutional Review Board of Jazan General Hospital provided ethical approval for this study.

## Results

A total of 1,718 participants completed the questionnaire with an 86.3% response rate. Of these, 1,554 were examined for IBS criteria, while 174 participants were excluded for either a self-reported medical history of organic diseases or alarming symptoms. Table 1 displays the sociodemographic characteristics of the study group. Men comprised 55% of the study group, and approximately 70% were under 40 years old. Most of the study population were university graduates (72%), with almost half of them working in the governmental sector. Gender and educational levels showed a statistically significant association with IBS (p-value < 0.05). 

**Table 1:** Sociodemographic characteristics of study group and associations with irritable bowel syndrome * = significant association (p-value < 0.05)

Variable	Frequency	Percent (%)	P-value
Gender (n = 1554)			0.011*
Male	860	55.3%	
Female	694	44.7%	
Age (n = 1554) (years)			0.605
18-29	654	42.1%	
30-39	497	32.0%	
40-49	310	19.9%	
50-59	75	4.8%	
60-69	18	1.2%	
Marital status (n = 1554)			0.112
Single	555	35.7%	
Married	955	61.5%	
Divorced	30	1.9%	
Widowed	14	0.9%	
Career (n = 1554)			0.985
Governmental sector	710	45.7%	
Private sector	145	9.3%	
Student	369	23.7%	
Unemployed	330	21.2%	
Educational level (n = 1554)			0.027*
Illiterate	2	1.0%	
Less than high school	59	3.8%	
High school graduate	313	20.1%	
University graduate	1119	72.0%	
Advance academic degree	61	3.9%	
Salary per month (Saudi Riyal) (n = 1554)			0.992
Less than 5000	477	30.7%	
From 5000 to 10000	460	29.6%	
From 10000 to 15000	407	26.2%	
More than 15000	210	13.5%	
Residence (n = 1554)			0.220
Jizan	247	15.9%	
Sabya	287	18.5%	
Abo Arish	261	16.8%	
Samtah	266	17.1%	
Faifa	245	15.8%	
Baish	248	16.0%	

The prevalence of IBS based on the Rome IV criteria among the study population was 16% (Table 2). IBS was more prevalent in females than males (18.5% and 13.8%, respectively). Among IBS subtypes, mixed (32.66%) and constipation-predominant (32.25%) subtypes were dominant.

**Table 2:** Prevalence of IBS and its subtypes IBS, irritable bowel syndrome; IBS-C, constipation predominant; IBS-D, diarrhoea predominant; IBS-M, mixed type; IBS-U, unspecified type

IBS Prevalence, n (%)							
IBS			Non-IBS			Total	
248 (16%)			1306 (84%)			1554	
Male	Female		Male	Female		Total Male 860	Total Female 694
119 (13.8%)	129 (18.5%)		741 (86.2%)	565 (81.5%)			
IBS Subtypes, n (%)							
IBS-C		IBS-D	IBS-M		IBS-U	Total	
80 (32.25%)		65 (26.2%)	81 (32.66%)		22 (8.9%)	248	

Table 3 illustrates the association between multiple behavioural and psychological risk factors with IBS using bivariate analysis. Khat consumption did not show a significant association with IBS, while coffee consumption, tobacco smoking, stress, and anxiety had a statistically significant (p-value < 0.05) association with IBS. Additionally, around 30% of the IBS population experienced stress or anxiety.

**Table 3:** Association between irritable bowel syndrome (IBS) and risk factors n, number. * = significant association (p-value < 0.05)

Variable	IBS, n (%)	Non-IBS, n (%)	P-value
Khat consumption			0.745
Yes	86 (15.5%)	467 (84.5%)	
No	162 (16.1%)	839 (83.9%)	
Caffeinated product consumption			0.048*
No	82 (17.8%)	379 (82.2%)	
1-2 cups/daily	113 (13.8%)	704 (86.2)	
3 cups or more/daily	53 (19.2%)	223 (80.8%)	
Tobacco smoking			0.007*
Yes	60 (21.2%)	222 (78.8%)	
No	188 (14.8%)	1084 (85.2%)	
Stress			0.000*
Yes	123 (31%)	274 (69%)	
No	125 (10.8%)	1032 (89.2%)	
Anxiety			0.000*
Yes	137 (27.8%)	356 (72.2%)	
No	111 (10.5%)	950 (89.5%)	

Table 4 shows the multiple regression analysis performed for selected IBS risk factors. Female gender (odds ratio (OR) = 1.503, p-value = 0.037), stress (OR = 2.386, p-value = 0.000), anxiety (OR = 1.943, p-value = 0.000), and tobacco smoking (OR = 2.093, p-value = 0.001) significantly predicted the presence of IBS. Women were 1.5 times more likely to experience IBS than men. In addition, participants with psychological risk factors including stress and anxiety were 2.3 and 1.9, respectively, times more likely to have IBS. Lastly, tobacco smokers were two times as likely to have IBS compared to non-smokers.

**Table 4:** Multiple regression analysis for selected irritable bowel syndrome (IBS) risk factors β, regression coefficient; SE, standard error; OR, odds ratio; CI, confidence interval. * = significant association (p-value < 0.05)

Variables	β (SE)	P-value	OR	95% CI (OR)	
				Lower	Upper
Gender (female)	0.408 (0.196)	0.037*	1.503	1.024	2.206
Educational level (illiterate)	Reference.				
(less than high school)	-1.530 (1.486)	0.303	0.216	0.012	3.984
(high school)	-2.233 (1.461)	0.126	0.107	0.006	1.878
(university graduate)	-2.140 (1.455)	0.141	0.118	0.007	2.037
(advance academic degree)	-1.663 (1.488)	0.264	0.190	0.010	3.506
Caffeinated product consumption (no consumption)	Reference.				
(1-2 cups/daily)	-0.229 (0.166)	0.167	0.795	0.574	1.101
(3 cups or more/daily)	0.062 (0.207)	0.764	1.064	0.709	1.597
Stress (Yes)	0.870 (0.179)	0.000*	2.386	1.681	3.387
Anxiety (Yes)	0.664 (0.177)	0.000*	1.943	1.372	2.751
Khat consumption (Yes)	-0.035 (0.221)	0.874	0.966	0.627	1.488
Tobacco smoking (Yes)	0.739 (0.216)	0.001*	2.093	1.370	3.199

## Discussion

This study revealed an overall IBS prevalence of 16% among the Jazan population in Saudi Arabia. To our knowledge, this is the first study among this specific population to explore the prevalence of IBS and its associated factors using an Arabic translated and validated questionnaire. 

The prevalence of IBS in this study appears to be higher than the global IBS rate based on the Rome IV criteria, which has an average rate of 4% [7,8]. However, the data from Middle Eastern countries regarding IBS prevalence is limited. The different methodologies used to collect this data with cultural differences may play a role in the prevalence variability [8,9]. For example, Egypt, as a middle-eastern country involved in the Rome Foundation Global Study, showed the highest IBS (Rome IV) prevalence (7.6%) in comparison to other countries [7].

Locally, in the Kingdom of Saudi Arabia, our results are consistent with the range of IBS prevalence reported in previous studies, which was between 9% and 40% [11,12,14-16,24]. Studies conducted using older versions of Rome criteria (i.e., Rome II and III) showed a higher prevalence rate than recent studies that used the Rome IV criteria. Recent studies in Saudi Arabia using the latest Rome criteria (Rome IV) mainly involved Saudi undergraduate students and board-certified physicians. They reported prevalence rates of 15.8% and 16.3% of IBS, respectively, which is similar to our study finding (16%) [15,16]. This decline in IBS prevalence was also obvious through multiple global studies, which is most likely due to the changes made in the diagnostic criteria of IBS. These changes included substituting the abdominal “discomfort” term with “pain” instead. This change raised the minimum pain threshold and the pain relation to defecation, and made a major impact on this decrease in the IBS rate globally and locally [5,6].

Regarding the IBS subtypes, IBS-M (mixed subtype) and IBS-C (constipation subtype) were predominant in this study, which is similar to the findings of two previous studies in Saudi Arabia [11,25]. IBS-M (mixed subtype) seems to be the most common subtype, according to recent IBS epidemiological data [26].

In the present study, women were 1.5 times more likely to be affected with IBS than men, similar to many studies [18]. According to several Western countries and Saudi Arabia reports, IBS is more prevalent in women than in men [16,27,28]. However, many studies from other Asian countries have revealed no gender differences in IBS prevalence [28].

Regarding age, our results did not show a significant association between age groups and IBS prevalence, which contrasts with the findings of most IBS prevalence studies [18,26]. The reason for this difference could be because majority of our study subjects were relatively young. In addition, older age groups that were determined to have IBS in our study may have had undetected organic diseases causing their symptoms instead. 

In the current study, cigarette smokers were two times more likely to have IBS than non-smokers. This is consistent with a local study, which investigated the prevalence of IBS among Saudi undergraduate students [16]. Although the relationship between smoking and IBS is inconsistent, no significant association could be confirmed based on a systematic review of data from 50 articles to determine the smoking frequency and association among patients with IBS [19].

Psychological risk factors play a major role in predisposing individuals to develop IBS [20,29]. The results of the present study revealed that one out of every three IBS-categorized participants had either stress and/or anxiety, with a statistically significant association with IBS (p-value < 0.05). Similarly, three local studies in Saudi Arabia reported higher stress and anxiety among IBS patients with a significant association [11,16,25]. Stress, anxiety, and high levels of catecholamines disrupt the haemostasis of brain-gut interactions. Moreover, as with all functional GI disorders, brain-gut interaction dysregulation tends to alter gut motility, visceral sensitivity, release of neuropeptide hormones, and gut microbiota, which finally lead to the presentation of IBS [30-33]. 

Khat (Catha edulis) is a chewable green leaf that is highly addictive and commonly chewed in Jazan Region [34]. Khat has been linked to an increased risk of depression, anxiety, and stress [35]. In addition, it was found to affect the GI system with several disorders through multiple observational studies, but none of these studies demonstrated a clear relationship between khat consumption and IBS [36,37]. In our study, there was no significant association between khat consumption and IBS. 

Although the present study provided valuable information about the prevalence of IBS and its associated risk factors in the study population, it still has some limitations. Participants who met the IBS diagnostic criteria in our cross-sectional study were not examined to rule out other possible disorders. Another limitation is that using a web-based cross-sectional study rather than the traditional method may have affected this study's response rate, particularly since illiterate or individuals living in areas without internet could not participate in the study sample. However, internet usage has become an integral part of most Saudi general populations, and many local studies have been conducted through web-based platforms [15,16,38,39]. Moreover, the cross-sectional methodology used in this study could not establish a causation between IBS and the risk factors. Then again, the strengths of this study included using validated and Arabic-translated Rome IV questionnaire, using a random sampling technique to minimize selection bias, and inclusion of all Jazan region sectors to generate a representative sample of the actual Jazan population. The current study will undoubtedly enrich the epidemiological data regarding IBS prevalence (using the latest Rome criteria) in Saudi Arabia. 

## Conclusions

The current study revealed an IBS prevalence rate of 16% in the south-west region of Saudi Arabia. IBS was significantly associated with female sex, tobacco smoking, and mental health disorders, mainly stress and anxiety. Our findings will provide insight regarding the impact of IBS on our community. Future studies should explore the causal relationship between IBS and its associated risk factors in Saudi Arabia.
